# Targeting pathogenic immune cells in rheumatoid arthritis: emerging immunotherapeutic frontiers

**DOI:** 10.3389/fimmu.2026.1895048

**Published:** 2026-10-05

**Authors:** Yan Dong, Xifang Xu, Sathyamoorthi S, Jindong Dai, Vijayakumar Murugesan, Selvaraj Muthusamy, Kanagaraj Rajalakshmi, Dongwei Zhu

**Affiliations:** 1 Department of Immunology, Jiangsu Key Laboratory of Laboratory Medicine, School of Clinical Medicine & Laboratory Medicine, Jiangsu University, Zhenjiang, China; 2 Department of Laboratory Medicine, Yizheng People’s Hospital, Yangzhou, China; 3 Department of Orthopaedics, Swamy Vivekanandha Medical College Hospital and Research Institute, Elayampalayam, Tamil Nadu, India; 4 Department of Pharmaceutics, School of Pharmacy, JSS Academy of Higher Education and Research, Vacoas, Mauritius; 5 Department of Immunology, Jiangsu Key Laboratory of Laboratory Medicine, School of Medicine, School of Chemistry and Chemical Engineering, Jiangsu University, Zhenjiang, China

**Keywords:** cytokine inhibitors, immune cells, immunotherapy, monoclonal antibodies, rheumatoid arthritis

## Abstract

Rheumatoid arthritis (RA) is a persistent autoimmune condition that predominantly affects the joints resulting in inflammation, pain and progressive disability. Immunological imbalance, especially the stimulation of pathogenic immune cells, including T and B cells, macrophages, amongst others, is the cause of the disease, as it is critical to the development of the disease. The limitation of traditional anti-RA medications, such as non-steroidal anti-inflammatory drugs (NSAIDs) and disease-modifying anti-rheumatic drugs (DMARDs), in the treatment of the disease led to the development of more specific medicines. One of the most promising biologic agents, including monoclonal antibodies, cytokine inhibitors and cell-based therapies, are specifically targeting immune cells and inflammatory pathways in RA. This review examines these new immunotherapies, their mechanisms of action, clinical efficacy, and challenges associated with them, including resistance to treatment, safety issues, and the necessity to use personalised therapy. Also, the new immunotherapeutic approaches, which include targeting autoantibodies, gene therapy, and precision medicine, are promising new approaches that could result in more effective and personalised treatments. Finally, the review concludes with a discussion of future directions, where in combination therapies, next-generation biologics, and personalised treatment regimens are also possible and can change the way RA can be managed in the future. To preserve analytical depth, this review explicitly distinguishes established (regulatory-approved) therapies from investigational and experimental approaches and indicates the level of clinical evidence for each.

## 1 Introduction

Rheumatoid arthritis (RA) is an autoimmune disorder of a multifactorial nature, which is marked by chronic inflammation and destruction of the joints (1–4). Around 1 percent of the world population is affected by it, amongst other women the prevalence is greater. The mechanisms of pathogenesis of RA include dysregulation of the immune system, which results in chronic inflammation of the synovium that subsequently leads to the destruction of cartilages and bones (5–7). T cells, B cells, macrophages, and dendritic cells are the major pathogenic immune cells in RA and all of them take part in the inflammatory process by different means including releasing cytokines, activating autoantibodies, and the development of the rheumatoid factor (8) (**Figure 1**).

**Figure 1:**
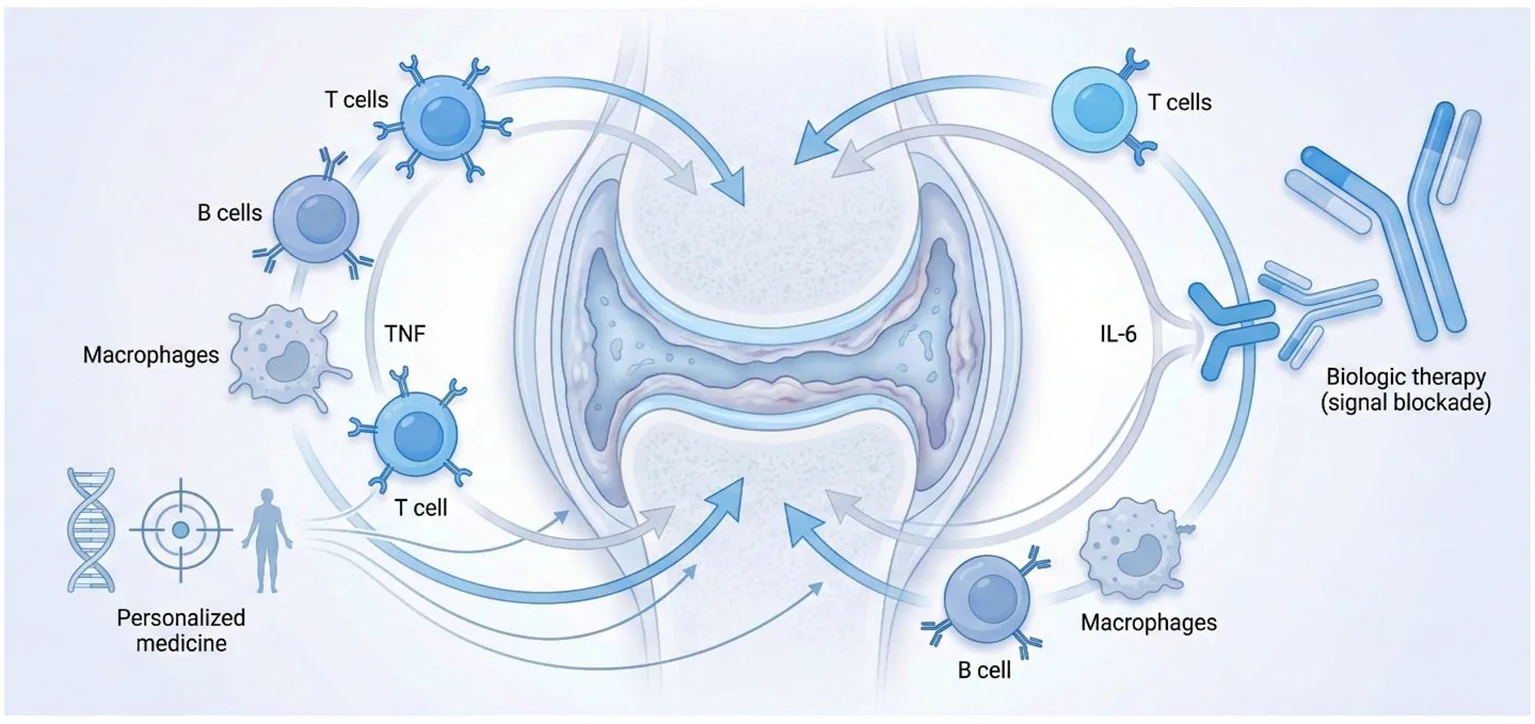
This figure illustrates the major immune cells involved in rheumatoid arthritis (RA) pathogenesis, including T cells, B cells, macrophages, and dendritic cells. It also depicts the role of cytokines such as TNF and IL-6 in driving inflammation and how targeted therapies, like TNF inhibitors and B-cell depletion, are used to modulate these immune responses.

The immune system plays a central role in RA pathogenesis. Genetically predisposed individuals are believed to react to the environment, by getting infected or smoking, to initiate the first wave of the immune response (9–11). This step is followed by the T cells and B cells activation that results in the synthesis of pro-inflammatory cytokines such as tumour necrosis factor (TNF), interleukin-6 (IL-6), and interleukin-1 (IL-1), which contribute to the inflammation and tissue damage in the joints (12). These cytokines stimulate the action of the macrophages and the development of the pannus, a bulk of inflammatory tissue, which attacks the joint and destroys cartilage and bone (13, 14).

### 1.1 Immunopathogenesis of rheumatoid arthritis: contemporary mechanisms

Contemporary understanding of RA pathogenesis extends well beyond broad immune-cell categories to a network of cellular and molecular endotypes that increasingly guide therapy. Genetic susceptibility is dominated by HLA-DRB1 shared-epitope alleles, which shape the presentation of citrullinated self-peptides and underlie anti-citrullinated protein antibody (ACPA) production (1, 9). Accumulating evidence indicates that ACPA responses are first generated at mucosal sites-particularly the periodontium, lung and gut-years before clinical synovitis, supporting a mucosal-origins hypothesis of RA (15–18), consistent with broader evidence linking gut-derived metabolites to mucosal immune dysregulation in other chronic inflammatory conditions (3).

Within the inflamed joint, an imbalance between pro-inflammatory T-helper 17 (Th17) cells and immunosuppressive regulatory T cells (Tregs) sustains synovitis, whilst T follicular helper (Tfh) cells provide B-cell help and drive autoantibody maturation within ectopic lymphoid structures that form in the synovium (1, 19, 20). Fibroblast-like synoviocytes (FLS) are now recognised as active, aggressive effectors rather than passive bystanders: they degrade extracellular matrix, secrete IL-6 and chemokines, and help define distinct synovial pathotypes (lymphoid, myeloid and fibroid) that correlate with treatment response (1, 21).

Neutrophils contribute through the release of neutrophil extracellular traps (NETosis), a key source of citrullinated autoantigens that perpetuate ACPA-driven inflammation (22). Immune complexes engage Fcγ receptors and activate the complement cascade, amplifying synovial inflammation, whilst receptor activator of nuclear factor-κB ligand (RANKL), produced by activated T cells and FLS, drives osteoclastogenesis and the bone erosion characteristic of established disease (23–27). Recognition of these mechanisms-increasingly captured by synovial immunophenotyping and molecular endotyping-now informs the rational, stratified selection of the immunotherapies discussed below.

The existing RA therapeutic measures, which mainly include non-steroidal anti-inflammatory drugs (NSAIDs) and traditional disease-modifying anti- rheumatic drugs (DMARDs) are meant to manage inflammation and reduce disease progression (28). This is however not the case in all patients and these treatments have serious side effects. Rituximab, TNF inhibitors, and IL-6 inhibitors are biologic DMARDs that have changed the treatment of RA over the last few years. These biologic agents are specific to immune cells or cytokines in the disease process and are better in terms of efficacy and minimal side effects (29).

In spite of these successful treatments, there are still problems, such as incomplete response of some patients, safety, and expensive nature (30). Besides, the heterogeneous character of RA implies that there might be a need to treat patients in a more individual manner (31). At this, new immunotherapies, such as novel biologics, targeted small molecules, and cell-based therapies are the future of the RA treatment. This review will concentrate on these new immunotherapies, the mechanisms of action of the immunotherapies, clinical trial results and future perspectives (32) (**Table 1**).

**Table 1:** This table summarizes the key immune cell types involved in RA pathogenesis, their activation mechanisms, cytokines released, their role in the inflammatory process, targeted therapies, and expected therapeutic outcomes in managing RA.

Immune cell type	Activation mechanism	Cytokines released	Role in RA pathogenesis	Targeted therapies	Therapeutic outcome
T Cells	Activation via Antigens	IL-1, TNF, IL-6	Drives inflammation and joint damage	TNF inhibitors, IL-6 inhibitors	Reduced disease activity
B Cells	Autoantibody Production	RF, ACPA	Contribute to immune complex formation	Rituximab, B-cell depletion	Decreased autoantibody levels
Macrophages	Cytokine Release	TNF, IL-1, IL-6	Inflammation and joint destruction	TNF inhibitors, IL-1 inhibitors	Reduced joint inflammation
Dendritic Cells	Antigen Presentation	IL-12, IL-6	Stimulate T cell response	Tolerogenic DCs	Modulation of immune tolerance
Tregs	Immune Suppression	TGF-β, IL-10	Prevents autoimmunity	Treg-based therapies	Improved immune regulation
Synovial Fibroblasts	Cytokine Production	IL-6, TNF	Leads to pannus formation	JAK inhibitors	Reduced pannus formation

## 2 Review methodology

This article is a narrative (non-systematic) mini-review. Relevant literature was identified through structured searches of PubMed/MEDLINE, Scopus and Web of Science covering January 2015 to 2025, supplemented by seminal earlier publications and by hand-searching the reference lists of key reviews. Search terms combined MeSH and free-text keywords including “rheumatoid arthritis”, “immunotherapy”, “biologic DMARD”, “monoclonal antibody”, “cytokine inhibitor”, “JAK inhibitor”, “cell-based therapy”, “regulatory T cells”, “mesenchymal stem cells” and “precision medicine”. English-language, peer-reviewed primary studies, randomised controlled trials, systematic reviews and authoritative narrative reviews were eligible; conference abstracts, non-peer-reviewed material and unpublished data were excluded. Because this is a narrative synthesis rather than a systematic review, no formal meta-analysis or risk-of-bias scoring was undertaken; the evidence was appraised qualitatively, and regulatory-approved therapies are distinguished from investigational and experimental approaches throughout, with the supporting evidence consolidated in **Table 2**.

**Table 2:** Safety, clinical status, efficacy and risk-management profile of immune-targeted therapies.

Therapeutic class/representative agents	Clinical status & major indications	Evidence level	Major efficacy findings	Key safety concerns	Pre-treatment screening	Monitoring/risk management
TNF inhibitors – Infliximab, Adalimumab, Etanercept, Certolizumab, Golimumab	Established/approved biologics; widely used in RA, IBD, PsA, AS and other inflammatory diseases	High – extensive RCTs and long-term real-world evidence	Rapid and sustained reduction in inflammatory activity; improves clinical response, remission and functional outcomes	Serious/opportunistic infections, TB reactivation, hepatitis B reactivation, injection/infusion reactions; possible malignancy signal	TB testing (IGRA/TST ± chest radiography), HBV/HCV screening; vaccination review	CBC, liver function and infection surveillance; avoid live vaccines during significant immunosuppression
IL-6 receptor inhibitors – Tocilizumab, Sarilumab	Approved biologics for RA and selected inflammatory conditions	High – RCTs and post-marketing data	Strong suppression of inflammatory activity; effective in patients with inadequate response to conventional DMARDs or TNF inhibitors	Neutropenia, elevated transaminases, dyslipidaemia, serious infections and lower-GI perforation	TB, HBV/HCV screening; baseline CBC and liver enzymes; assess GI risk factors	Periodic CBC, ALT/AST and lipid profile; monitor for infection and abdominal symptoms suggestive of GI perforation
B-cell depletion – Rituximab	Established biologic; approved for RA and several autoimmune/haematological indications	High – RCTs and extensive clinical experience	Effective disease control through sustained B-cell depletion; useful after inadequate response to TNF inhibitors	Infusion reactions, serious infections, hypogammaglobulinaemia, late-onset neutropenia and HBV reactivation; impaired vaccine response	HBV/HCV and TB screening; baseline immunoglobulins; vaccination before treatment where feasible	CBC and immunoglobulin levels; monitor infections and HBV reactivation; consider antiviral prophylaxis in appropriate HBV-risk patients
JAK inhibitors – Tofacitinib, Baricitinib, Upadacitinib	Approved targeted synthetic DMARDs for selected inflammatory diseases	High – RCTs plus extensive long-term safety surveillance	Rapid improvement in disease activity and symptoms; effective in patients with inadequate response to conventional/biologic DMARDs	MACE, VTE, serious infection including herpes zoster, malignancy and cytopenias; important regulatory safety warnings	TB, HBV/HCV screening; cardiovascular/VTE risk assessment; baseline CBC and lipids	CBC, liver enzymes and lipid profile; monitor cardiovascular, thrombotic and infectious complications; individualise use in high-risk patients
IL-1 receptor antagonist – Anakinra	Approved biologic for selected autoinflammatory and inflammatory disorders	Moderate–High – RCTs and clinical experience	Reduces IL-1-mediated inflammation and improves disease manifestations in responsive conditions	Injection-site reactions, neutropenia and increased infection risk	TB/HBV/HCV assessment according to clinical risk; baseline CBC	CBC and infection surveillance; avoid live vaccines during immunosuppression
T-cell co-stimulation modulator – Abatacept	Approved biologic for RA and selected inflammatory arthritides	High – RCTs and long-term evidence	Sustained improvement in disease activity, physical function and structural outcomes in RA	Serious infections, infusion/injection reactions and possible respiratory complications; generally favourable safety profile	TB and viral hepatitis screening; vaccination review	CBC, clinical infection surveillance and periodic assessment for respiratory/infectious complications
Biologic therapies – immunogenicity consideration	Applies particularly to therapeutic monoclonal antibodies, especially chimeric antibodies	Moderate–High – immunogenicity and pharmacovigilance evidence	Anti-drug antibodies may contribute to secondary loss of response and treatment failure	Anti-drug antibodies (ADAs), reduced drug exposure, loss of efficacy and infusion reactions	Review previous biologic exposure and treatment history	Therapeutic-drug monitoring and ADA testing when clinically indicated; concomitant methotrexate may reduce immunogenicity
All immune-targeted therapies – infection prevention framework	Applies across biologic and JAK-targeted therapies	High – guideline/consensus and pharmacovigilance evidence	Risk reduction through appropriate screening, vaccination and early infection management	Opportunistic infection, TB and viral reactivation	TB (IGRA/TST ± chest radiography), HBV/HCV screening and vaccination assessment	Periodic infection assessment; complete indicated vaccines before immunosuppression where possible; latent TB requires appropriate preventive therapy
Overall risk-management framework	Individualised selection based on disease severity, comorbidities, previous treatment response and infection/cardiovascular risk	High – integrated clinical evidence and safety surveillance	Maximises benefit–risk balance through efficacy-guided and safety-guided treatment selection	Class-specific toxicities, immunosuppression, infection, cytopenias, hepatic abnormalities and cardiovascular/thrombotic events	Comprehensive baseline clinical assessment, TB/HBV/HCV screening, CBC, liver enzymes, lipids and vaccination review	Regular clinical review, laboratory monitoring, infection vigilance and class-specific surveillance; reassess treatment benefit versus cumulative risk

ADA, anti-drug antibody; AS, ankylosing spondylitis; CBC, complete blood count; DMARD, disease-modifying antirheumatic drug; GI, gastrointestinal; HBV, hepatitis B virus; HCV, hepatitis C virus; IBD, inflammatory bowel disease; IGRA, interferon-γ release assay; JAK, Janus kinase; MACE, major adverse cardiovascular events; PsA, psoriatic arthritis; RA, rheumatoid arthritis; TB, tuberculosis; TNF, tumour necrosis factor; TST, tuberculin skin test; VTE, venous thromboembolism.

## 3 Cell-based therapeutics in rheumatoid arthritis

The cell-based therapies are considered to be an effective method of treating rheumatoid arthritis (RA), and their aim is to regulate the immune response by transplanting various types of cells (33). These medications are used to restore or modify the immune system to eliminate inflammation and avoid the destruction of joints. It is an overview of the best investigated cell-based therapies in RA: stem cells, tolerogenic dendritic cells (DCs), regulatory T cells (Tregs), and comments on the difficulties and the success of these therapies in clinical trials (**Figure 2**). It should be emphasised that, in contrast to the regulatory-approved biologic and small-molecule agents discussed in later sections, all cell-based strategies in RA remain investigational; they are supported predominantly by preclinical data and small early-phase trials and are therefore presented here as emerging rather than established therapies.

**Figure 2:**
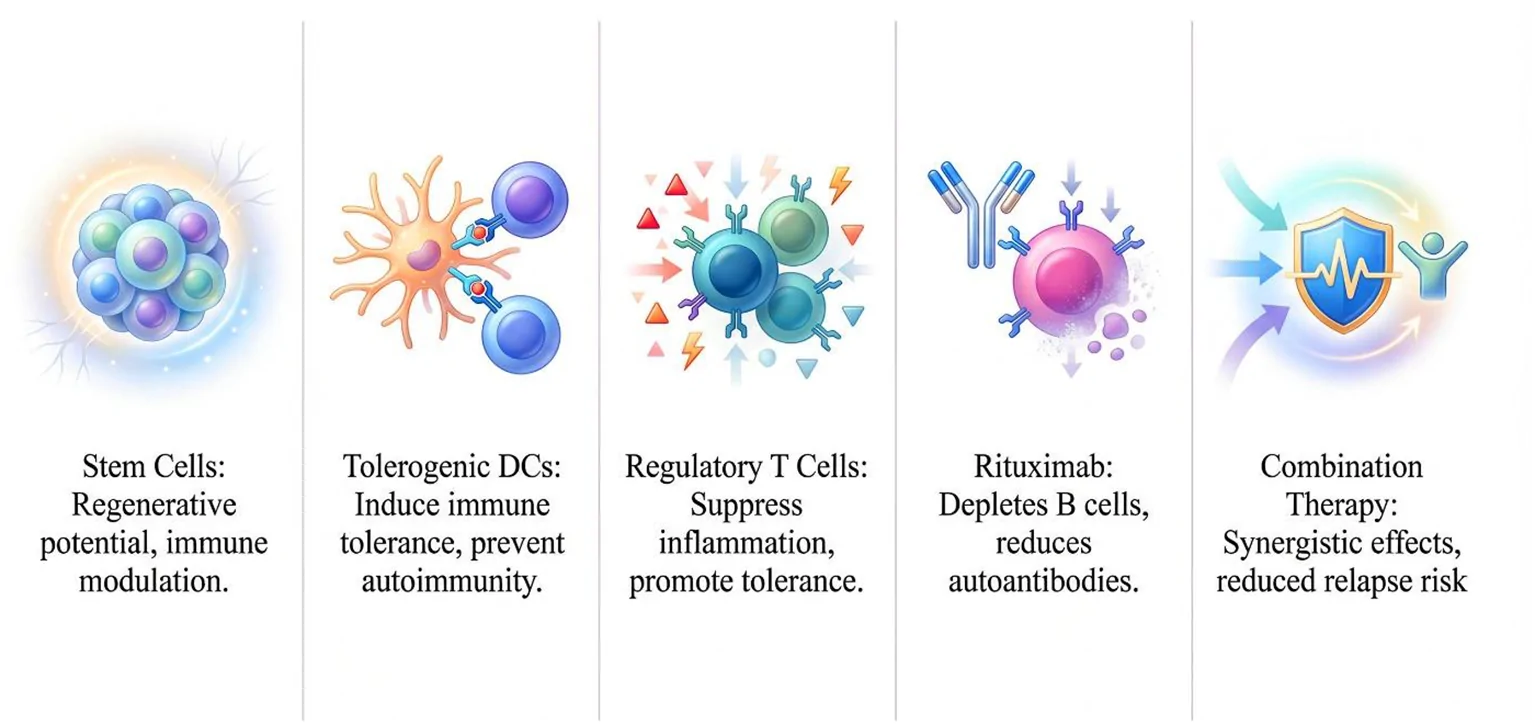
Overview of cell-based therapies for RA, showcasing stem cells, tolerogenic dendritic cells, regulatory T cells, and B cell depletion therapy, along with their mechanisms, clinical applications, and challenges.

### 3.1 Stem cells and RA treatment

There is a great potential in the use of stem cell therapy in the treatment of RA particularly because of its regenerative capability (34). The stem cells are undifferentiated cells which have the potential to self-renew and differentiate into other cell types, including the immune-modulating cell types. Mesenchymal stem cells (MSCs) are amongst the many types of stem cells that have had the most potential in RA treatment. MSCs may be obtained through various sources amongst them being bone marrow, adipose tissue and umbilical cord blood. They have immunomodulatory functions i.e. the inhibitory effect on the activation of T cells, B cells, and macrophages, which are major contributors to the pathogenesis of RA (35).

MSCs have a number of interactions with the immune system. They also release soluble factors such as transforming growth factor- beta (TGF-β) and prostaglandin E 2 (PGE2) that are capable of suppressing T cell growth and lowering the production of inflammatory cytokines. Moreover, MSCs are suggested to stimulate the repair of the tissues as they are capable of differentiation into chondrocytes and osteoblasts, which help to repair cartilage and bone. This regenerative potential is what makes them especially attractive as treatment of RA where the destruction of joints is one of the symptoms of the disease (36).

There has been a promising response of early clinical trials that could be used to examine the application of MSCs in RA, especially in the suppression of disease activity and amelioration of clinical symptoms. In one instance, a study proved that patients with RA who underwent allogeneic MSC treatment had significant decrease in the disease activity scores (DAS28) and had functional improvement. Nevertheless, the safety and efficacy of MSC-based therapy are still under research and issues like the optimal source of MSCs, delivery procedures, and long-term consequences should be resolved before these types of therapy can be used widely in clinics (37).

### 3.2 Tolerogenic dendritic cells

Dendritic cells (DCs) are significant in the process of induction and control of immunity (38). DCs in RA help to stimulate autoreactive T cells, which carry on with the inflammatory cascade. Amongst the most promising strategies of cell-based therapy of RA is the application of tolerogenic DCs that are designed to trigger immune tolerance as opposed to immune activation.

Tolerogenic DCs can be engineered or naturally occurring DCs capable of inhibiting immune responses. Those cells can develop regulatory T cells (Tregs), which inhibit the work of the pathogenic T cells and prevent autoimmunity. The idea behind tolerogenic DCs in RA would be to re-establish the immune system to accept self-antigens without the development of inflammation. These DCs may be obtained in different sources and then subjected to immunosuppressive factors e.g., dexamethasone or rapamycin to be transformed into a tolerogenic phenotype (39).

Although limited preclinical research and preliminary clinical trials have indicated that tolerogenic DCs can be used in the treatment of RA (40). According to a research conducted, the tolerogenic DCs were capable of evoking a regulatory immune response in an autoimmune disease patient, such as RA. Tolerogenic DC therapy with clinical trials has demonstrated a reduction in the activity of the disease and an improvement in the functioning of the joints. Nevertheless, there are still problems, especially in the aspect of the production and stability of these cells. Moreover, the best protocol relating to the production and administration of tolerogenic DCs is yet to be discovered.

### 3.3 Regulatory T cells

Tregs are T cells which are also known as regulatory T cells which are important in the preservation of immune tolerance and prevention of autoimmune responses (19). The Tregs also tend to be lacking or malfunctioning in RA, which is a factor that leads to uncontrolled activation of the effector T cells and consequent inflammatory mechanisms. In this regard, Tregs restoration or growth has become a possible approach to RA treatment.

Tregs suppress the immune system in several ways, amongst them, by secreting anti-inflammatory cytokines, including IL-10 and TGF-β, or by directly inhibiting the activation of effector T cells (41). RA Tregs can either be expanded *in vitro* and reintroduced into patients or provoked *in vivo* with the help of different cytokines and small molecules. The latter, particularly as regulated by Tregs, is of interest because they are able to regulate the immune system without generally weakening it and may circumvent the side effects of the conventional immunosuppressive drugs.

A number of studies have been done on the utilisation of ex vivo-expanded Tregs in RA. An interesting study by Teng et al. revealed that the administration of autologous Tregs clones derived out of patients with RA resulted in a significant decrease in the level of disease activity and the production of inflammatory cytokines. Furthermore, the Treg therapy seemed to be safe and there were no severe side effects stated (42). Nevertheless, it is unclear how Treg-based treatments are effective and stable in the long run. The difficulty in producing enough numbers of Tregs *in-vitro* and the possibility of Treg dysfunction in severe cases of RA are some of the challenges. Moreover, selective growth of Tregs and not development of other T cell subsets is a major challenge.

### 3.4 Issues and clinical trials

Although the cell-based therapies in RA have given promising results, there are still a number of challenges in the clinical use of it. The heterogeneity of RA as such is one of the major issues (1). RA is a multifactorial disease that is a complex one and the responses to cell-based therapies may differ greatly between patients. The duration of the disease, severity and the autoantibodies that are present may also determine the effectiveness of these remedies. In addition, there is still controversy as to when intervention is best done, when cell-based therapies are most effective in one country, at very early stages of the disease or at more advanced stages when tissue damage has already occurred. Accordingly, the current level of evidence for these approaches is low, deriving largely from small, often single-arm Phase I/II studies, and none has yet achieved regulatory approval for RA.

The other issue is the issue of safety and sustainability of cell-based therapies in the long-term. Although the short term results of stem cell therapy, tolerogenic DCs, and Treg infusion have been favourable in initial clinical trials, the long term implications of the practise are yet to be realised (43). The possibility of tumorogenesis, infection, or antigen rejection of transplanted cells must be taken care of by subjecting them to stringent clinical studies. Moreover, the technical problems with manufacturing and quality control of these cells, its cost and accessibility are also serious obstacles in the universal clinical implementation of the technology.

There are also clinical trials in place to explore cell-based therapies in treating RA and most of them involve the safety and efficacy of MSCs, tolerogenic DCs, and Tregs. As an example, a Phase I/II trial (NCT02200320) is analysing the safety and effectiveness of MSC therapy in patients with RA, initial outcomes indicate an inflammatory and disease activity reduction. On the same note, clinical trials examining Treg therapy (NCT01818432) are also looking into whether ex vivo-expanded Tregs can eliminate disease symptoms and also prevent joint destruction (44). These trials however are still in their infancy and before these therapies can be prescribed in normal clinical practise, the outcomes of such trials must be confirmed through larger and long-term studies.

Also, the use of cell-based therapies with already existing treatments, including biologics or small molecules, is an actively investigated field. A combination therapy can have synergistic effect where the combined therapy’s effectiveness is increased and the risk of resisting or relapsing is less (45) (**Table 3**).

**Table 3:** Summary of cell-based therapies in rheumatoid arthritis, highlighting various cell types, their mechanisms of action, sources, immunomodulatory functions, clinical trial outcomes, and challenges faced in their clinical implementation.

Cell type	Mechanism of action	Source	Immunomodulatory function	Clinical trials	Challenges
Mesenchymal Stem Cells (MSCs)	Immunomodulation, tissue repair	Bone marrow, adipose, umbilical cord	Inhibits T/B cells, macrophages; promotes tissue repair	Significant decrease in disease activity	Long-term safety, optimal source, delivery issues
Tolerogenic Dendritic Cells (DCs)	Induces immune tolerance	Various sources	Develops Tregs, suppresses autoimmune responses	Preliminary studies show reduced disease activity	Stability, production challenges
Regulatory T Cells (Tregs)	Suppresses immune responses	Autologous or expanded ex vivo	Secretes anti-inflammatory cytokines (IL-10, TGF-b)	Significant decrease in disease activity	Difficulty in expansion, selective growth issues
B Cell Depletion (Rituximab)	Depletes autoantibody-producing B cells	Infusion therapy	Reduces autoantibody levels, improves joint function	Effective in TNF-inhibitor non-responders	Potential reactivation of infections
Combination Therapy	Synergistic effects with other therapies	Combination of various agents	Enhances effectiveness, reduces relapse	Trials with MSCs and Tregs underway	Safety and dosing concerns
Clinical Trial Issues	Safety, efficacy, and long-term studies	Various trials (e.g., NCT02200320)	Focus on disease suppression and prevention of joint destruction	Early-phase trial results positive	Heterogeneity of RA, cost and accessibility

## 4 Monoclonal antibodies in RA therapy

Monoclonal antibodies (mAbs) have transformed the treatment paradigm of rheumatoid arthritis (RA) with its highly specific form of treatment provided by inhibiting such critical elements of the immune system that are involved in the pathogenesis of the disease (46). Since autoimmune diseases such as RA are mediated by the immune cells activation and release of inflammatory cytokines, mAbs have been effective in terms of addressing these elements in order to minimise inflammation and avoid destruction of the joints. In this section, it has been summarised the currently most frequently used monoclonal antibodies in the treatment of RA, including TNF-blockers, IL-6-blockers, rituximab, and new antibodies (**Figure 3**).

**Figure 3:**
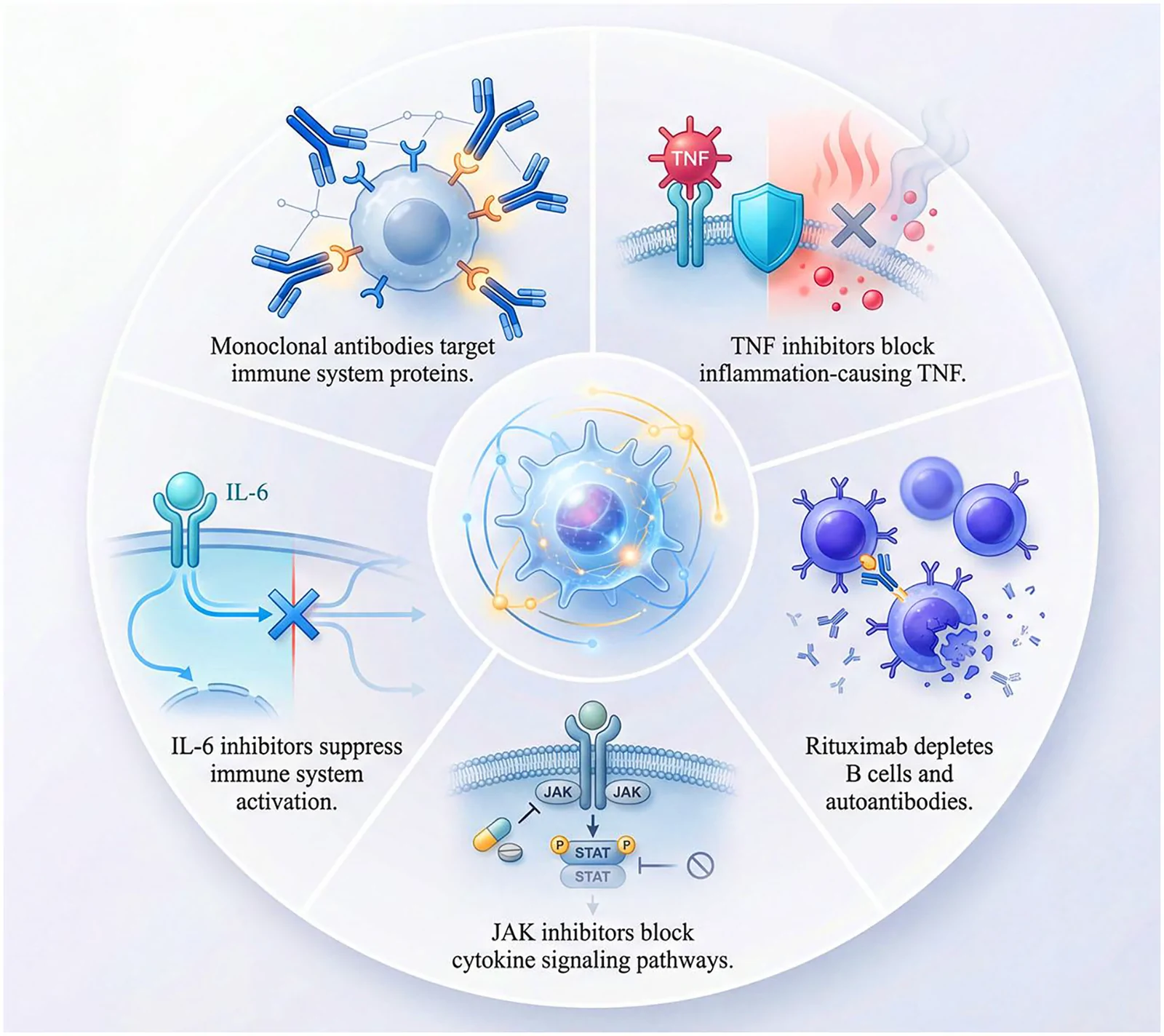
This figure illustrates the role of monoclonal antibodies in RA therapy, highlighting their targets, mechanisms, and clinical efficacy. It emphasizes the importance of TNF, IL-6, and JAK inhibition in disease management.

### 4.1 TNF inhibitors

Essential in the pathogenesis of RA is tumour necrosis factor-alpha (TNF-α) which is a pro-inflammatory cytokine (47). It is secreted by several types of immune cells such as Macrophages, and it is instrumental in the onset and the spread of inflammation in RA by creating other cytokines, such as IL-1, and IL-6. TNF-α causes the activation of matrix metalloproteinases (MMPs) and eventual degradation of cartilages when it is elevated in the synovium.

TNF inhibitors such as infliximab, etanercept, adalimumab and golimumab have become the new mainstay treatments of moderate to severe RA. These biologics inhibit the TNF-α signalling either by binding the cytokine (infliximab, adalimumab) or by serving as soluble TNF-α receptors (etanercept) (48). TNF inhibitors have been shown to promote clinical activity in RA by reducing the disease activity, joint destruction, and systemic inflammatory activity and enhancing physical functioning and quality of life.

Nevertheless, even though the TNF inhibitors are effective, they are not effective in all patients, and in certain cases, they are resistant to treatment or they cause adverse effects including infections, infusion reactions or increase risk of malignancy (49). These shortcomings have necessitated the need to identify more therapies that have alternative mechanisms of action.

### 4.2 IL-6 inhibitors

The other cytokine which is central to RA is interleukin-6 (IL-6) which facilitates inflammation, activates acute-phase reactants as well as mediating B cells and T cells differentiation. The IL-6 is increased in the synovial fluid in the patients with RA and it leads to the inflammation and destruction of the joints (50).

The discovery of IL-6 inhibitors like tocilizumab has created another treatment solution to the patients with RA especially those who did not respond well to TNF inhibitors. Tocilizumab is a humanised monoclonal antibody binding to IL-6 receptor, which is an IL-6 signalling inhibitor (51). It has shown strong effectiveness in lowering the activity of the disease as well as the outcome of RA patients such as those with high concentrations of inflammatory cytokines such as C-reactive protein (CRP) and those who do not respond to other biologics.

Besides tocilizumab, other newer IL-6 inhibitors, including sarilumab, have also been constructed with equal mechanisms of action. It is demonstrated that both tocilizumab and sarilumab reduce the symptoms of joint inflammation and enhance physical functioning and prevent structural damage in patients with RA (52). Nevertheless, similarly to the TNF inhibitors, the IL-6 inhibitors are linked to side effects, such as the greater susceptibility to infection, liver enzyme abnormalities, and neutropenia.

### 4.3 Rituximab and B cell depletion

Rituximab is a chimeric monoclonal antibody that attacks surface protein CD20 that is present on B cells (53). Rituximab is a chimeric (murine/human) anti-CD20 monoclonal antibody that binds CD20 and leads to the depletion of B cells by promoting the antibody-dependent cell-mediated cytotoxicity and complement-dependent cytotoxicity. The effect of this depletion is the loss of circulating autoantibodies, such as rheumatoid factor (RF) and anti-citrullinated protein antibodies (ACPAs) that have been linked to the pathogenesis of RA.

Rituximab has been demonstrated to be useful in the treatment of RA especially where the TNF inhibitors fail to work. Rituximab has a positive clinical effect, namely, enhanced disease activity, less joint damage and better functional outcomes (54). It can be combined with methotrexate to increase its effectiveness and minimise the chances of the relapse.

Rituximab is not without adverse effects despite its effectiveness. The side effects were most noticeable, as infusion reactions or the possibility of the infection (with the possible reactivation of hepatitis B) and the long-term consequences on humoral immunity (55). Moreover, not all patients can be guaranteed recovery without B cell repletion, which means that the remission of RA can be relapsed.

### 4.4 Other novel targeted agents (non-antibody biologics and small molecules)

For accuracy of biologic-drug classification, it is important to note that several agents frequently grouped with “biologics” are not monoclonal antibodies. Abatacept is a soluble CTLA-4–Ig fusion protein (not a monoclonal antibody), and the Janus kinase (JAK) inhibitors tofacitinib, baricitinib and upadacitinib are targeted synthetic small molecules rather than antibody-based agents. Amongst the agents discussed below, only golimumab is a true (fully human) anti-TNF-α monoclonal antibody. These distinctions are reflected in the classification given in **Table 2**. Over the past years, several new targeted agents directed against various elements of the immune system have been developed, which offer more alternatives to the treatment of RA. Some of these include:

- Abatacept - Abatacept is a T-cell co-stimulant inhibitor that consists of a fusion protein, consisting of the CTLA-4 and the Fc of human IgG1, blocking the interaction of CD80/86 between antigen-presenting cells to CD28 on T cells. This inhibits the T cell-activation and the subsequent inflammatory cascade. Abatacept has shown to be effective in TNF inhibitor-naive patients as well as TNF inhibitor-experienced patients of RA (56).
- Janus kinase (JAK) inhibitors: Although not monoclonal antibodies, oral tofacitinib and baricitinib inhibitors of the Janus kinase pathways (JAKs) represent intracellular signalling pathways, which activate immune cells (57, 58). They inhibit the JAK-STAT axis, which is essential in cytokine signalling in RA. The JAK inhibitors form a potential substitute to patients who cannot get biologic injections.
- Golimumab: Golimumab is a human monoclonal antibody targeting TNF-α, although it is as much as other TNF inhibitors, it is delivered less often (once every 8 weeks), because of the long half-life (59) This is convenient to the patients who do not need to be treated frequently. These newer targeted agents are able to reach a wider spectrum of components of the immune system and provide more treatment options to RA patients whose condition was not affected by conventional regimes. These newer agents are still undergoing assessment on clinical trials although they have shown promise they still need more testing to determine their safety and effectiveness in the long term.

### 4.5 Clinical outcomes and mechanisms of action

The way of action of monoclonal antibodies in treating RA is different according to which antibody is the target. The TNF inhibitors act by blocking the receptor of TNF-α to the immune cells and the further inflammatory cascade (60). On the other hand, IL-6 inhibitors prevent the association of IL-6 with its receptor, which inhibits the triggering of inflammatory signalling pathways. Rituximab causes the depletion of B cells and thus suppresses the generation of autoantibodies and also suppresses the inflammatory reaction.

Monoclonal antibody therapy has had a great impact on the clinical outcomes of the RA disease (61). All of the TNF inhibitors, the IL-6 inhibitors and rituximab have shown improvements in the disease activity, the physical functioning and the minimum in structural damage in the clinical trials. These therapies along with the resulting reduction of the disease activity scores (DAS28) and the results of the health assessment questionnaire (HAQ) scores mark the efficacy of the therapies (62). Moreover, there are biomarkers like CRP and ESR to measure the effect of therapy, mAb therapies showed a good effect in lowering the intensity of inflammation.

Monoclonal antibodies have safety profiles that are mostly acceptable although infusion reactions, side effects like infections and hematologic abnormalities should be closely monitored. The long term safety is an issue due to the risk of malignancies and severe infections that are increased (63). Consequently, individualised regimens of treatment as per the patient features, such as comorbidities and history of previous treatment are fundamental in streamlining the application of monoclonal antibodies in RA (24) (**Table 4**).

**Table 4:** Summary of monoclonal antibodies used in rheumatoid arthritis (RA) therapy. Includes targets, mechanisms of action, indications, clinical efficacy, and potential side effects observed in clinical trials.

Monoclonal antibody	Target cytokine/protein	Mechanism of action	Indication	Efficacy	Side effects
TNF Inhibitors	Tumor Necrosis Factor (TNF)	Binds TNF, preventing its signalling	Moderate to severe RA	Reduces inflammation, joint damage	Infections, infusion reactions, malignancy risk
IL-6 Inhibitors	Interleukin-6 (IL-6)	Blocks IL-6 receptor, inhibiting IL-6 signalling	RA unresponsive to TNF inhibitors	Reduces disease activity and CRP	Infections, liver enzyme abnormalities
Rituximab	CD20 on B cells	Depletes B cells, reducing autoantibody production	RA unresponsive to TNF inhibitors	Decreases disease activity, joint damage	Infusion reactions, infection risk
Abatacept	CD80/86, T cells	Inhibits T cell activation by blocking co-stimulation	TNF inhibitor-naive or experienced RA	Reduces inflammation, disease activity	Infections, hypersensitivity reactions
JAK Inhibitors	Janus Kinases (JAK)	Inhibits JAK-STAT pathway, blocking cytokine signalling	Moderate to severe RA	Reduces inflammation, improves joint function	Infections, blood clots, liver enzyme issues
Golimumab	Tumor Necrosis Factor (TNF)	Binds TNF, preventing its signalling	Moderate to severe RA	Effective, less frequent administration	Infections, infusion reactions, malignancy risk

## 5 Cytokine-targeted therapies

The cytokines are small signalling proteins which are crucial in mediating the effects of inflammation and immune response in rheumatoid arthritis (RA) (1). One of the components contributing to the chronic inflammation observed in RA is the dysregulation of cytokine networks, which result in the damage of the joints and functional disability (21, 64). The application of cytokine-targeted therapy that aims at blocking pro-inflammatory cytokines, including interleukin-1 (IL-1), interleukin-6 (IL-6), and Janus kinases (JAKs) has become a part and parcel of the RA treatment (65). These treatments are more precise and focused compared to conventional DMARDs, which have a much greater positive clinical outcome in most patients (66). The review presented in this section provides a detailed overview of the most popular cytokine-targeted therapies in RA (67)(**Figure 4**).

**Figure 4:**
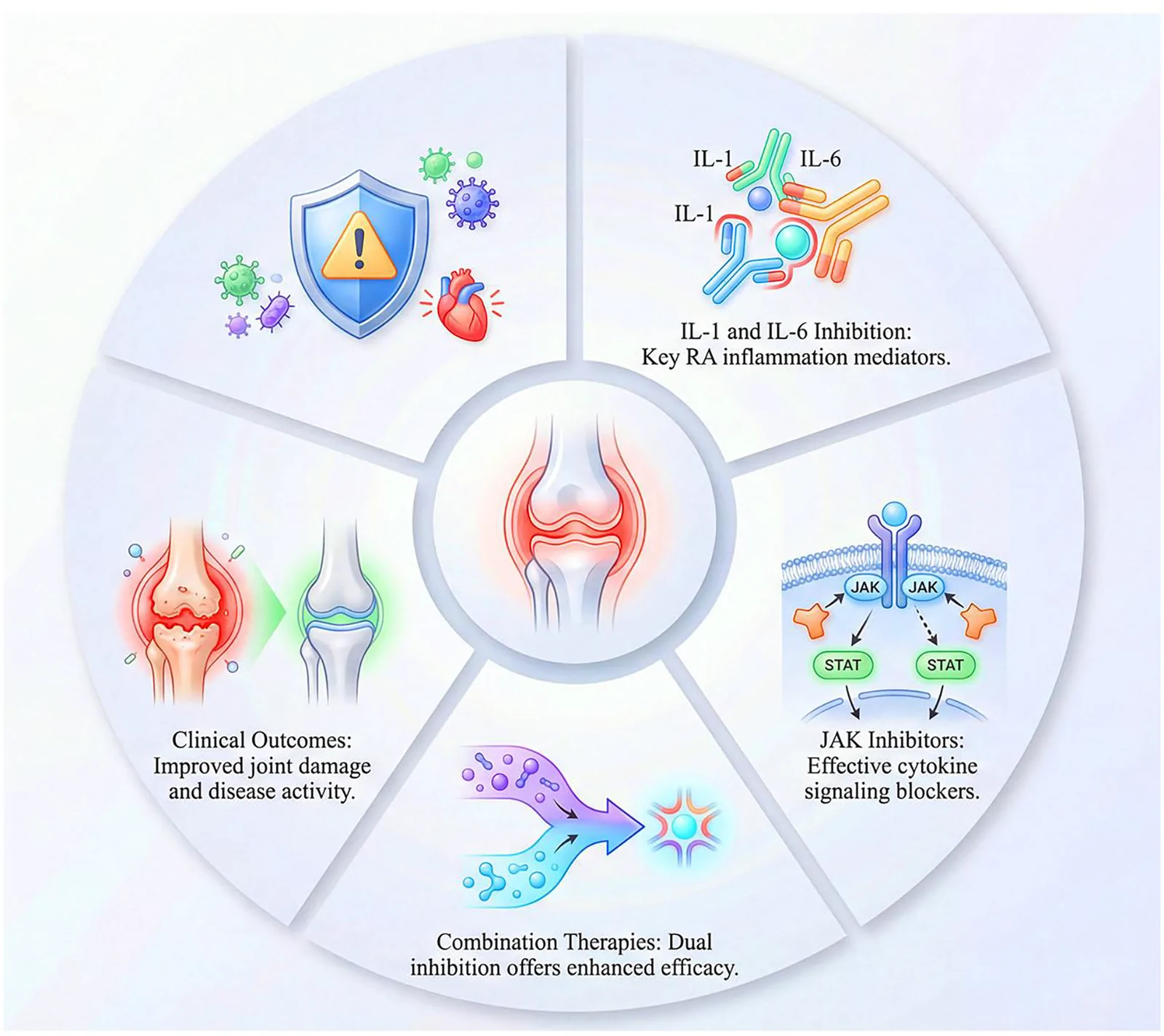
This table summarizes key cytokine-targeted therapies used in rheumatoid arthritis (RA), detailing their mechanisms of action, efficacy, administration methods, and associated safety concerns.

### 5.1 Targeting IL-1 and IL-6 pathways

Important pro-inflammatory cytokines that mediate the pathogenesis of RA include IL-1 and IL-6 (68, 69). The effect of IL-1 is mainly connected with the early development of the inflammation and activation of immune cells, although IL-6 is important in the promotion of the systemic inflammation, stimulation of the production of the acute-phase response proteins, and the differentiation of B cells. The two cytokines are involved in sustaining an inflammatory condition in RA and inhibition of their signal transduction pathways has been useful in the management of inflammation and symptom flare-up in patients with RA.

Anakinra is also an IL-1 inhibitor that has been employed to prevent the activation of the IL-1 receptor (70). Anakinra is a recombinant version of IL-1 receptor antagonist (IL-1Ra), which displaces IL-1 as the binding agonist to IL-1 receptor so that its activity is inhibited. Though anakinra has been proven to be effective in managing clinical symptoms of RA, its application has been hampered by the need to inject patients subcutaneously on a daily basis, and relatively low efficacy in contrast to other biologic agents such as TNF. This lower comparative efficacy has been shown in randomised head-to-head data rather than assumed by analogy, and is the principal reason anakinra is now used mainly as a second-line option once TNF-, IL-6- or JAK-targeted therapy has failed or is contraindicated.

The other important cytokine in RA is IL-6 and its targeting, IL-6 signalling, is emerging as an effective approach to treatment (71). Tocilizumab is an IL-6 receptor antagonist and has been used in the treatment of RA and it has demonstrated great effectiveness in the reduction of the disease activity. Tocilizumab lowers systemic inflammation, suppresses T cell differentiation, and osteoclast differentiation by inhibiting the IL-6 signal. Tilizumab is shown to be safe and effective in clinical trials to reduce joint damages and physical abilities in patients with RA when combined with methotrexate (72, 75).

### 5.2 JAK inhibitors

Small molecules of Janus kinase (JAK) inhibitors block intracellular communication of various cytokine cascades in RA pathogenesis (73–75). JAKs are cytokine mediating enzymes which include IL-6, IL-2, IL-4, and IL-15. JAKs are crucial in facilitating the inflammatory effect and destruction of the joints in RA.

Tofacitinib, baricitinib and upadacitinib are the most popular JAK inhibitors used in RA. These medications selectively suppress JAK1, JAK 2 and JAK3 to interfere with the signalling of pro-inflammatory cytokines. The first JAK inhibitor approved in RA is tofacitinib that was found to be quite effective in the reduction of the disease activity and in terms of clinical outcomes both in monotherapy and in combination with methotrexate (68, 76). The efficacy of baricitinib and upadacitinib has also been demonstrated to be favourable in clinical trials, but baricitinib has some more advantages compared to upadacitinib including quick action and prolonged efficiency.

The use of JAK inhibitors is a significant advancement in the management of RA since this can be administered orally with a wide range of cytokine suppression. Nevertheless, they cannot be utilised without being associated with risks since they may increase individuals susceptibility to infections, blood clots, and possible liver enzyme abnormalities (70, 77). The safety data are under study and optimal usage over a long period with the aid of other therapies is part of a research area.

### 5.3 Dual targeting therapies

To have a more holistic and effective anti-inflammatory effect, dual targeting therapies are meant to inhibit two or more pathways of inflammation at the same time (78). In RA, such treatments aim at using a combination of cytokine and JAKs/other molecular target inhibitors to deal with the multi-complexity and two-sidedity of the disease (79).

The IL-6-JAK combination is one of the examples of dual targeting therapy. Such a combination strategy acts on IL-6 signalling pathway and JAK-STAT signalling pathway, all of which play a central role in the inflammatory process in RA (80). There have been encouraging outcomes of this combination therapy with regards to a clinical trial at the early phases as it offers a stronger decrease in disease activity as well as improvement in clinical aspects than a monotherapy.

The other possible approach is through the use of biologics which address both TNF and IL-6 (81). It has been proved in preclinical studies that the combined inhibition of the two cytokines can result in synergistic effect causing greater inflammation suppression and protecting joint destruction. There is, however, a need to conduct more studies to determine the safety and effectiveness of this kind of combination therapies in large clinical trials (82). It should be stressed that dual-targeting strategies remain investigational, the IL-6-JAK and combined TNF/IL-6 approaches described above are supported only by early-phase or preclinical data, and none currently holds regulatory approval for RA, in contrast to the single-target biologics and JAK inhibitors summarised in **Table 2**.

### 5.4 Clinical effectiveness and safety issues

Cytokine-based interventions, such as IL-1 and IL-6-blockers, and JAK-inhibitors have offered an important contribution to the RA treatment (83). These treatments provide specific and successful treatment procedures to patients who fail to respond to conventional DMARDs. Nevertheless, there are not so many difficulties in utilising these therapies.

Possibility of severe infections is one of the major issues. IL-6 and JAK inhibitors suppress immune system and predisposes the person to opportunistic infections including fungi and tuberculosis. During the use of these biologics, close follow-up of patients to identify infection symptoms is required (84). Also, the JAK inhibitors are linked to an elevated risk of thrombosis and cardiovascular events, and their long-term safety is a matter of concern.

Cytokine-targeted therapies are also a big problem in the cost. Biologics and small molecule inhibitors are commonly costly and their expensive nature can make these therapies very unattainable to the majority of patients, especially in the low and middle-income nations.

Though this is the case, cytokine-targeted therapies continue to play a key role in the management of RA in the contemporary world. Further studies on combination therapies, and better monitoring of their safety will allow reducing the dangers of these types of treatment and their further positive effect on clinical results (**Table 5**).

**Table 5:** This table summarizes key cytokine-targeted therapies used in rheumatoid arthritis (RA), detailing their mechanisms of action, efficacy, administration methods, and associated safety concerns.

Therapy	Cytokine targeted	Mechanism of action	Administration route	Clinical efficacy	Safety concerns
Anakinra	IL-1	IL-1 receptor antagonist; inhibits IL-1 activity	Subcutaneous injection	Reduces RA activity, inflammation	Daily injection, limited efficacy
Tocilizumab	IL-6	IL-6 receptor antagonist; inhibits IL-6 signalling	Intravenous injection	Effective for inflammation, damage	Infection risk, liver enzyme issues
Tofacitinib	JAKs (JAK1, JAK3)	Inhibits JAK-mediated cytokine signalling	Oral administration	Reduces disease activity, joint damage	Infection risk, thrombosis
Baricitinib	JAKs (JAK1, JAK2)	JAK inhibitors; suppresses pro-inflammatory cytokines	Oral administration	Rapid effect, prolonged efficacy	Infections, cardiovascular concerns
Upadacitinib	JAKs (JAK1)	Selective JAK1 inhibition; reduces cytokine release	Oral administration	Reduces disease activity, relieves pain	Long-term safety concerns
Dual Targeting	IL-6, JAKs	Combination of IL-6 receptor blockade & JAK inhibitors	Combination therapy	Stronger effect than monotherapy	Safety, potential for serious infections

## 6 Small molecule and innovative immunotherapies

The last few years have seen the rise of the use of small molecules and the creation of innovative immunotherapies as a new option in treating RA. The objectives of these therapies are to be much more specific in targeting underlying disease-causing molecular mechanisms, and may provide more effective and less toxic alternatives to conventional therapies (85–88). The sub-sections will touch on emerging small molecules, ones that target autoantibodies, those that employ nanoparticle-based drug delivery systems, and those that employ peptide-based immunomodulation (89–92).

### 6.1 Targeting autoantibodies

Rheumatoid factor (RF) and anti-citrullinated protein antibodies (ACPA) are examples of autoantibodies that are characteristic features of RA, and serve to drive pathologic processes by connecting with the immune complexes, which trigger inflammatory systems (1). Attacking such autoantibodies has become a potential approaach, which could help to stop the progression of RA and avoid joint destruction.

B-cell depletion therapy that includes rituximab has been applied in order to decrease the production of autoantibody. Rituximab attaches to CD20 of B cells causing their depletion and consequent decrease in the level of autoantibodies. This method has worked in other patients who have refractory RA and are not responding to other biologics (16). Nevertheless, the B-cell depletion long-term outcomes and the possibility of reactivation of the infections are also aspects that need to be taken into consideration.

New treatments are underway in specific ways to focus on the production of autoantibody (93, 94). Amongst the methods, the targeting of certain B-cell signal transduction mechanisms, including Bruton tyrosine kinase (BTK), an important B-cell activation factor, has been used. BTK inhibition can help prevent the occurrence of autoantibodies, as well as optimise inflammation in RA (17, 22). Initial phase clinical trials on BTK inhibitors have provided encouraging outcomes and more research is being conducted to determine the safety and effectiveness of these drugs on RA patients (25). Rituximab-based B-cell depletion is the only autoantibody-directed strategy discussed here with regulatory approval for RA; BTK inhibition and other autoantibody-production-blocking approaches remain investigational, supported so far only by early-phase trials.

### 6.2 Nanoparticle-based drug delivery

The drug delivery systems that have been developed in recent years as nanoparticles have attracted a lot of attention because they enhance the pharmacokinetic and bioavailability of therapeutic agents and minimise systemic side effects (1). It is possible to create nanoparticles to carry drugs and target them to the area of inflammation to increase efficacy of treatment and reduce off-target effects.

The measurement of nanoparticle-based systems to deliver biologic agents, small molecules, and anti-inflammatory drugs to the target is being investigated in RA (94). As an example, TNF inhibitors, IL-1 antagonists, or JAK inhibitors can be encapsulated in liposomes or polymeric nanoparticles, which can release the encapsulated molecules in the inflamed joints at a sustained rate. This specific method may result in better treatment and may decrease the frequency of dosage, which makes it a promising way of enhancing patient compliance and quality of life (25). Nanoparticle-encapsulated formulations for RA are, at present, experimental: reported data are chiefly preclinical or early-phase, no such formulation is yet regulatory-approved for RA, and delivered payload identity (e.g., an already-approved TNF inhibitor) should not be conflated with approval of the nanoparticle platform itself.

### 6.3 Peptide-based immunomodulation

Peptide-based therapy provides a new strategy of regulating the immune response in RA (95, 96). Such therapies are based on synthetic peptides which might act as agonists or antagonists of the action of certain immune molecules which play a role in the pathogenesis of RA. Indicatively, one can use the peptides of the protein citrullinated vimentin to prevent the interaction of autoantibodies with their respective antigens and hence the production of immune complexes and subsequent inflammatory reaction (97).

Immune tolerance can also be induced by designing a peptide based therapy, which would be useful in the treatment of autoimmune diseases such as RA (98, 99). This can be achieved through one of the methods, which is to use peptides that are specific to the T cells, and in so doing control them in order to induce differentiation of regulating T cells (Tregs) which inhibit the autoimmune response. Preliminarily, it has been demonstrated that peptide-based immunomodulation has the potential to significantly decrease the inflammatory state and damage to the joint in animal models of RA, and clinical trials are in progress to determine this in human patients (100). As with the other small-molecule and innovative approaches in this section, peptide-based immunomodulation remains preclinical-to-early-clinical and unapproved for RA; its evidence base is smaller and less mature than that of the approved biologic and JAK-inhibitor classes.

### 6.4 Progress in drug design and development

The development and designing of drugs has resulted in the development of drugs that are more selective and powerful in treatment of RA (28). Rapid identification of new drug targets and the design of small molecules that specifically modulate disease-causing pathways in RA is possible due to new technologies, including high-throughput screening, artificial intelligence, and computational modelling.

The development of selective inhibitors of particular cytokine pathways as in IL-17 or IL-23, which have been associated with the pathogenesis of RA, is also a promising field of drug development (101–103). These inhibitors are able to attack the disease processes at a more specific level, which may provide effective and safe ways of treatment as compared to the traditional therapies (67).

Conclusively, there are new small molecules and novel therapies with immunotherapy, which are reshaping the RA treatment (104). Autoantibody-targeted therapies that use nanoparticle-based delivery of drugs, and peptide-based immunomodulation have tremendous potentials towards offering patients with RA more effective, targeted, and personalised therapies. Further studies and research in the area should be conducted to further develop these therapies and enhance results of RA patients (100)(**Table 6**; **Figure 5**).

**Table 6:** This table summarizes emerging small molecules and innovative immunotherapies in RA treatment, highlighting their mechanisms of action, targeted molecules, advantages, challenges, and current research status.

Therapy	Mechanism of action	Targeted molecule/pathway	Advantages	Challenges	Current status
Targeting Autoantibodies	Depletes B cells, reduces autoantibody levels	RF, ACPA	Reduces inflammation, halts joint destruction	Long-term outcomes, infection risk	Ongoing clinical trials
Nanoparticle Drug Delivery	Encapsulates drugs in nanoparticles, targets inflamed joints	TNF inhibitors, JAK inhibitors	Increased drug efficacy, reduced side effects	High development cost, safety concerns	In preclinical/early trials
Peptide-Based Immunomodulation	Modulates immune response via synthetic peptides	T-cells, autoantibodies	Potential to induce immune tolerance, reduce inflammation	Efficacy and stability in long-term use	Early-phase studies
IL-17/IL-23 Inhibitors	Selectively inhibits IL-17 or IL-23 signalling	IL-17, IL-23	More targeted therapy, fewer side effects	Limited long-term data	Ongoing clinical trials
JAK Inhibitors	Inhibit JAK-STAT signalling pathways	IL-6, IL-2, IL-4	Oral administration, broad cytokine inhibition	Infections, thrombosis risk	Approved for use
Gene Therapy	Uses genetic modification to regulate immune cells	Immune cells	Potential for long-term remission	Technical, safety concerns	In development

**Figure 5:**
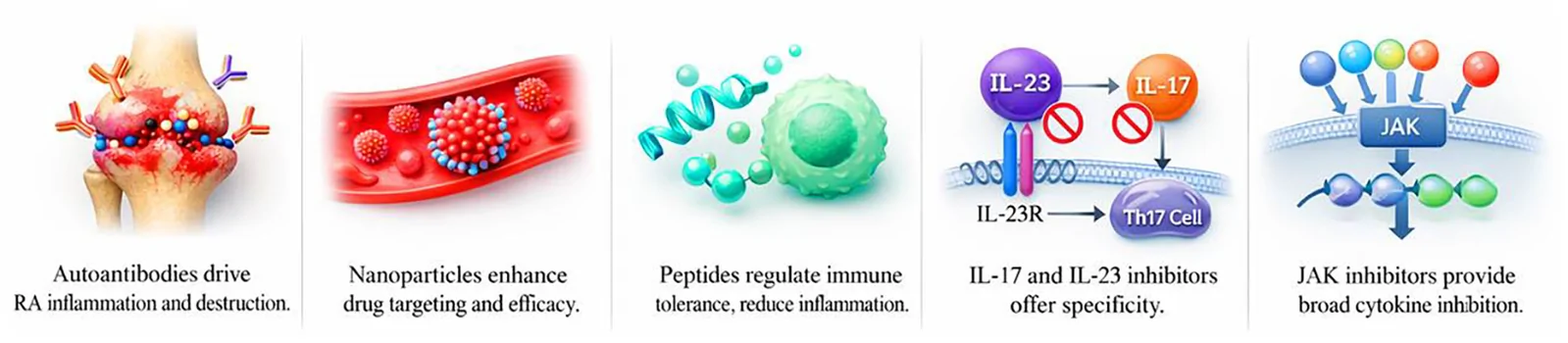
This manuscript offers a comprehensive review of the latest advancements in RA immunotherapy, providing valuable insights into emerging treatments, their mechanisms, and their clinical implications, setting the stage for personalized, effective therapies.

## 7 Safety considerations and risk management

Because immune-targeted therapies modulate host defence and inflammatory signalling, their benefits must be weighed against class-specific safety risks. The scattered safety information noted across the preceding sections is consolidated here into a single framework summarising the principal hazards and the screening and monitoring required across the major therapeutic classes; a class-by-class synthesis is provided in **Table 2**.

### 7.1 Infection risk and pre-treatment screening

All biologic and JAK-targeted therapies increase the risk of serious and opportunistic infection. Before initiation, patients should be screened for latent tuberculosis (interferon-γ release assay or tuberculin skin test with chest radiography) and for hepatitis B and C. Latent tuberculosis requires chemoprophylaxis before therapy, and hepatitis B carriers require antiviral cover to prevent reactivation-a concern of particular importance with the B-cell-depleting agent rituximab. Where possible, live-attenuated vaccines should be given before immunosuppression, and age-appropriate inactivated vaccination (influenza, pneumococcal and recombinant herpes zoster) should be brought up to date (60, 105).

### 7.2 Class-specific toxicities

TNF inhibitors carry risks of latent-infection reactivation, injection or infusion reactions and a debated association with malignancy. IL-6-receptor blockade (tocilizumab, sarilumab) is associated with neutropenia, transaminase elevation, dyslipidaemia (rises in LDL and HDL cholesterol that warrant lipid monitoring) and a risk of lower-gastrointestinal perforation. B-cell depletion with rituximab can cause hypogammaglobulinaemia after repeated cycles, late-onset neutropenia and impaired vaccine responses, in addition to hepatitis B reactivation. JAK inhibitors carry regulatory boxed warnings for major adverse cardiovascular events (MACE), venous thromboembolism (VTE), serious infection (including herpes zoster), malignancy and cytopenias, informed by long-term safety surveillance. Anakinra and abatacept are generally well tolerated but still elevate infection risk.

### 7.3 Immunogenicity and anti-drug antibodies

Biologic agents, especially chimeric antibodies, can elicit anti-drug antibodies (ADAs) that reduce efficacy and provoke infusion reactions. Concomitant methotrexate lowers immunogenicity and is therefore commonly co-prescribed; therapeutic-drug and ADA monitoring can help explain secondary loss of response (60).

### 7.4 Monitoring summary

In practice, safe use requires baseline tuberculosis and hepatitis screening with a vaccination review; periodic monitoring of the full blood count, liver enzymes and lipid profile; vigilance for infection and for thrombotic and cardiovascular events (notably with JAK inhibitors); and immunoglobulin monitoring with repeated B-cell depletion. These requirements are integrated with efficacy, regulatory status and indication data in **Table 2** to support balanced, individualised treatment selection (**Table 2**).

## 8 Future directions

The emerging vision of immunotherapy in rheumatoid arthritis (RA) is personalised medicine with its appropriate investment in genomics, proteomics and immunology. Individualised treatment plans would help define the unique patient profiles that would best respond to individualised treatment and enhance clinical outcome and reduce adverse events (2). This will be possible with help of the integration of genetic and molecular data in clinical decision-making. Other emerging biologic agents are those that appear to act on novel immune pathways like the inflammasome as well as microbiome. These pathways are important in the pathogenesis of RA and targeting them may offer novel therapeutic solutions to patients who do not respond to the existing treatment. Moreover, cell-based treatment, i.e. the application of mesenchymal stem cells (MSCs) and genetically engineered immune cells, are becoming new potential options to treat RA in the long-term. These treatments are also intended to inhibit inflammation as well as induce tissue repair and regeneration. When these novel techniques are united with precision medicine, one can even imagine a scenario when the future RA treatment will be based on the specific genetic and immunological characteristics of the individual patient, which will change the game of RA management and will lead to an increase in its positive consequences.

## 9 Discussion

Although biologic agents and targeted immunotherapies have increased the treatment outcomes of rheumatoid arthritis (RA) significantly, there remain a number of challenges. The high cost of biologic drugs is one of the biggest problems because it restricts their accessibility particularly in resource-bound environments. Also, these treatments may contribute to the risks of infections and other severe adverse events, which makes them difficult to use over time. Moreover, although numerous biologics have proven to be successful, not all the patients respond to the treatment in the same way, and some of them might develop resistance to treatment with time, which requires alternative or combination therapies. New specific cytokine, immune cell, or molecular pathway-targeted therapies hold promise of more useful solutions, but this area still needs to deal with these issues by designing better drugs, selecting patients better, and more vigilant safety monitoring. Since the study of the immune pathways underlying the RA remains ongoing, it can be anticipated that new treatment targets will be found that can provide us with a more tailored treatment. Individually designed therapies based on genetic and molecular data may have a chance to overcome resistance and enhance the response rate of a patient, which leads to an additional increase in the efficiency of RA therapies. Finally, a more refined and precision-based approach is the future of the RA treatment, and the current clinical trials and studies are essential in solving the existing shortcomings.

Set against one another, the therapeutic classes reviewed here differ markedly in maturity, effect size and risk profile, and these differences-summarised class-by-class in **Table 2**-should guide, rather than follow, treatment choice. TNF inhibitors and IL-6-receptor blockade have the deepest evidence base (multiple Phase III trials, long-term registry follow-up) and the most established safety signatures (infection, and for IL-6 blockade, dyslipidaemia and gastrointestinal perforation), making them the default biologic option after conventional-synthetic DMARD failure. Rituximab occupies a narrower but well-evidenced niche, particularly in seropositive and TNF-inhibitor-refractory disease, at the cost of humoral immune suppression. JAK inhibitors match TNF inhibitors on efficacy and offer oral dosing, but their boxed warnings for cardiovascular events, thromboembolism and malignancy mean the evidence, whilst extensive, now argues for more selective use in patients with pre-existing cardiovascular or thromboembolic risk. Abatacept and anakinra are supported by solid but comparatively modest efficacy data and are generally reserved for specific patient subgroups or as later-line options. By contrast, cell-based therapies (Section 3), dual-targeting combinations (Section 5.3) and the autoantibody-directed, nanoparticle-based and peptide-based strategies in Section 6 remain investigational: their supporting evidence is limited to small, often single-arm early-phase trials or preclinical models, none has regulatory approval for RA, and comparative safety data against established agents are largely absent. This gradient-from extensively validated biologics and JAK inhibitors, through agents with narrower but solid indications, to emerging approaches awaiting confirmatory trials-is the central evidentiary structure this review has tried to make explicit throughout, rather than treating all “immunotherapies” as equivalently supported.

## 10 Conclusion

To sum up, the introduction of immunotherapy in the management of rheumatoid arthritis (RA) has offered more selective and effective choices over conventional treatment. Small molecule or biologics inhibitors such as TNFs, IL-6s, and JAKs have played an important role in modifying the diseases and have brought relief to a large number of patients. Nevertheless, the discipline is still confronting some challenges such as excessive prices of biologics, the issue of long-term safety, and treatment resistance. With the further investigations of RA pathogenesis, new therapeutic approaches, such as cell-based ones or the targeting of the new immune pathways, are promising in further RA care development. Individualised treatment plans that are enabled by innovation in genomics and precision medicine are almost certain to become the foundation of the future of RA treatment, with a treatment being more productive and more tailored to the needs of a particular patient. Also, the evolution of combination therapy combining biologics, small molecules, and innovative methods of treatment can be proposed as a therapy that is more effective and has fewer side effects. With continuing clinical trials on the methods of improving existing treatment as well as testing new ones, the future of RA therapy is set to change dramatically. The problematic situation does not nullify the future of the treatment of RA, and new therapies have given some hope of better long-term control and quality of life in patients.
